# Title: Expansion of a national differentiated service delivery model to support people living with HIV and other chronic conditions in South Africa: a descriptive analysis

**DOI:** 10.1186/s12913-021-06450-z

**Published:** 2021-05-17

**Authors:** Lingrui Liu, Sarah Christie, Maggie Munsamy, Phil Roberts, Merlin Pillay, Sheela V. Shenoi, Mayur M. Desai, Erika L. Linnander

**Affiliations:** 1 Global Health Leadership Initiative, Yale School of Public Health, New Haven, USA; 2 Department of Health Policy and Management, Yale School of Public Health, New Haven, USA; 3 National Department of Health, Pretoria, South Africa; 4 Project Last Mile, Pretoria, South Africa; 5 Department of Medicine, Yale School of Medicine, Section of Infectious Diseases, AIDS Program, New Haven, USA; 6 Department of Chronic Disease Epidemiology, Yale School of Public Health, New Haven, USA

## Abstract

**Background:**

South Africa is home to 7.7 million people living with HIV and supports the largest antiretroviral therapy (ART) program worldwide. Despite global investment in HIV service delivery and the parallel challenge of non-communicable diseases (NCDs), there are few examples of integrated programs addressing both HIV and NCDs through differentiated service delivery. In 2014, the National Department of Health (NDoH) of South Africa launched the Central Chronic Medicines Dispensing and Distribution (CCMDD) program to provide patients who have chronic diseases, including HIV, with alternative access to medications via community-based pick-up points. This study describes the expansion of CCMDD toward national scale.

**Methods:**

Yale monitors CCMDD expansion as part of its mixed methods evaluation of Project Last Mile, a national technical support partner for CCMDD since 2016. From March 2016 through October 2019, cumulative weekly data on CCMDD uptake [patients enrolled, facilities registered, pick-up points contracted], type of medication provided [ART only; NCD only; and ART-NCD] and collection sites preferred by patients [external pick-up points; adherence/outreach clubs; or facility-based fast lanes], were extracted for descriptive, longitudinal analysis.

**Results:**

As of October 2019, 3,436 health facilities were registered with CCMDD across 46 health districts (88 % of South Africa’s districts), and 2,037 external pick-up points had been contracted by the NDoH. A total of 2,069,039 patients were actively serviced through CCMDD, a significant increase since 2018 (*p* < 0.001), including 76 % collecting ART [64 % ART only, 12 % ART plus NCD/comorbidities] and 479,120 [24 %] collecting medications for chronic diseases only. Further, 734,005 (35 %) of patients were collecting from contracted, external pick-up points, a 73 % increase in patient volume from 2018.

**Discussion:**

This longitudinal description of CCMDD provides an example of growth of a national differentiated service delivery model that integrates management of HIV and noncommunicable diseases. This study demonstrates the success of the program in engaging patients irrespective of their chronic condition, which bodes well for the potential of the program to address the rising burden of both HIV and NCDs in South Africa.

**Conclusions:**

The CCMDD program expansion signals the potential for a differentiated service delivery strategy in resource-limited settings that can be agnostic of the patients chronic disease condition.

## Background

In 2019, South Africa was home to 7.7 M people living with HIV (one in five adults), and supported the largest antiretroviral therapy (ART) program worldwide, with 4.8 M on ART [1]. South Africa also has a growing burden of non-communicable diseases (NCDs) including diabetes and hypertension, which account for 51 % of mortality and eclipsed the death rates from communicable diseases in 2016 [2–4]. As nations strive toward the UNAIDS 95-95-95 targets [5] and the 2030 Sustainable Development Goals, there is heightened interest in models of differentiated service delivery to ensure that health services and related medicines are available and accessible to those who need them, when and where they are needed most [6]. Further, there is a call to integrate HIV services with NCD care, maximizing resources, and addressing co-morbidities in tandem [7, 8].

While several models of differentiated service delivery (DSD) have been shown to reduce costs and improve the care for people living with HIV (PLHIV) [9–13], descriptive evaluations of DSD programs achieving national scale are lacking [14, 15]. Further, national programs offering multiple modalities of differentiated service delivery (both facility- and contracted, community-based; individual- and group-driven), that offer patients a choice in uptake, are now emerging [14–16]. Longitudinal evaluations of the path to scale for such programs would support evidence-informed global investment in scale-up and sustainabililty for DSD [17–19].

South Africa’s *“*National Guidelines for Adherence to HIV, TB, and NCDs” emphasizes addressing, among others, the structural barriers that patients face in accessing treatment and care [20]. The South African National Department of Health (NDoH) launched the Central Chronic Medicines Dispensing and Distribution (CCMDD) progamme in 2014 to support adherence and retention on ART by enabling patients to access their medicines from contracted, community-based pick-up points where they live and work [21]. The CCMDD programme is inclusive of the end-to-end solution which begins with the clinical review of a patient, dispensing of medications on the CCMDD formulary, delivery to points of service, and collection by patients via adherence clubs [20]; fast lanes at participating clinics; or external pick-up points located at independent pharmacies and participating retailers [21, 22]. It is a repeat prescription collection strategy offered to people with stable HIV and, importantly, stable chronic diseases.

In 2016, the NDoH began to catalyse the expansion of the program from the initial 11 pilot districts toward a national scale [11, 23]. This document presents a descriptive evaluation of this expansion over a 3.5 year period with increasing levels of detail as routine program monitoring systems were strengthened over time. These results will be useful to practitioners, policymakers, and implementers planning to scale-up innovative models of integrated service delivery, as well as to researchers evaluating and comparing DSD models.

## Methods

### Program description

The Central Chronic Medicines Dispensing and Distribution (CCMDD) progamme is positioned within the National Health Insurance (NHI) program and is intended to improve alternative access to medications for patients living with chronic disease including HIV and to address the demand associated with expanded access to ART envisaged under the Universal Test and Treat Guidelines implemented in late 2016 [23]. The program is available to patients who meet the eligibility criteria shown in Table 1 [24].

**Table 1:** Patient-level eligibility criteria for enrollment in CCMDD as of March 2018

Patients living with HIV who are on ART:
i. On same treatment regimen for at least 12 months
ii. Most recent lab results are normal
a. Most recent viral load result taken in past six months
b. 2 consecutive viral loads are undetectable
iii. Clinic confirms eligibility
a. Stable and adherent to treatment
b. No current TB
c. No medical conditions requiring regular clinical consultations
iv. Chronic medication items are on the provincial CCMDD medicine list
Patients with chronic non-communicable diseases:
i. For patients with diabetes – 2 consecutive fasting plasma glucose levels normal
ii. For patients with hypertension – 2 consecutive blood pressure readings normal

Eligible patients referred to the program, voluntarily register and select a pickup point (PuP) from which to collect their medication, returning to the health facility every 6 months for a clinical review and their prescription to be renewed. PuPs include contracted pick-up points (external PuP) in locations external to public health facilities, including independent pharmacies; community-based outreach clubs or adherence clubs; and fast lanes which are based at registered health care facilities. External PuPs are contracted by the NDoH to issue parcels of medication to participating patients. Parcels of medication are dispensed and distributed by two service providers who have a government tender to collect prescriptions; dispense and distribute medication throughout the eight participating provinces in South Africa (excludes Western Cape). Medication is provided by the provinces as per their medicine list. Participation in the program is free-of-charge to patients.

Project Last Mile is a global health public-private partnership that aims to strengthen health systems across Africa, evaluated using a longitudinal, mixed-methods design across country settings (as previously described [25]). Since 2016, The South African National Department of Health (NDoH) has worked in partnership with Project Last Mile, with funding from the United States Agency for International Development (USAID) to provide strategic support for CCMDD expansion. This has included (1) geomapping analytics to inform gaps in CCMDD coverage, (2) business case development to support national investment, (3) execution support to promote consistent PuP standards across the country, and (4) private sector engagement to support external PuPs and explore innovative modalities for CCMDD expansion.

### Measures of CCMDD programme expansion

We monitored CCMDD expansion from March 2016 through October 2019 (3 years, 7 months) using four measures: (1) the number of districts onboarded; (2) the number of health facilities registered; (3) the number of external pick-up points (external PuP) contracted; and (4) the number of patients registered. In January 2018, CCMDD began to differentiate the number of patients who had ever registered with CCMDD (registered patients) and the number of patients who were actively serviced (active patients). In November 2018, CCMDD began to collect more granular data through routine administrative systems, allowing us to differentiate active patients by (1) the type of medications that they were collecting (measured as ART, non-communicable disease, or a combination of ART and NCDs) and (2) patient’s choice of where medication parcels were collected (measured as external PuPs; outreach/ adherence clubs, and facility-based fast lanes).

### Data collection and ethics

This study is based on district-level administrative data of the CCMDD program from 2016 to 2019 across 8 provinces participating in CCMDD within South Africa: Eastern Cape, Free State, Gauteng, KwaZulu-Natal, Limpopo, Mpumalanga, Northern Cape, and North West (Western Cape is not participating), and includes all 46 districts of the 8 participating provinces.

The service providers of the CCMDD program provided weekly reports on patient volumes to the NDoH, managed in an Excel file for futher analysis. All data analysis procedures were approved by the South African National Department of Health (NDoH). As not human subjects research, the study was exempted from continuing review by the Human Subjects Committee of Yale University. This study was performed in accordance with the Declaration of Helsinki.

### Statistical analysis

Standard descriptive statistics were used to quantify the CCMDD programme expansion for the duration of PLM’s strategic support, from March 2016- October 2019. For each of the four measures, weekly district-level data was aggregated into monthly totals for the eight provinces participating in CCMDD programme. The changes over time were presented, with respect to the numbers of registered health care facilities, registered external PuPs, registered and active patients, and active patients by both their medication type and their preferred medication collection point. All analyses were performed using Stata SE15 (StataCorp, Texas) and Microsoft Excel (version 15.21.1).

## Results

**Fig. 1:**
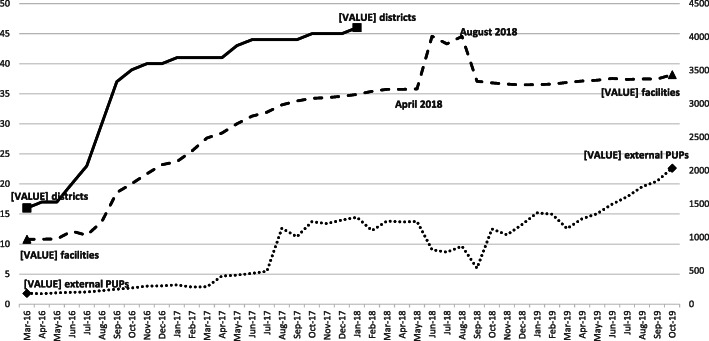
Number of Districts, Facilities, and External Pickup Points (external PuPs) Registered in the Central Chronic Medicines Dispensing and Distribution (CCMDD) Programme; from March 2016 through October 2019*

As shown in Fig. 1, CCMDD was operating in 16 districts at the start of March 2016, and expanded its reach to 46 districts from 8 participating provinces (88 % of health districts nationally) by January 2018. From March 2016 through October 2019, the number of health facilities increased from 972 to 3436 (representing 94.6 % of all facilities in the participating districts), and the number of external pick-up points increased 12-fold over the observation period, from 164 to 2037.

**Fig. 2:**
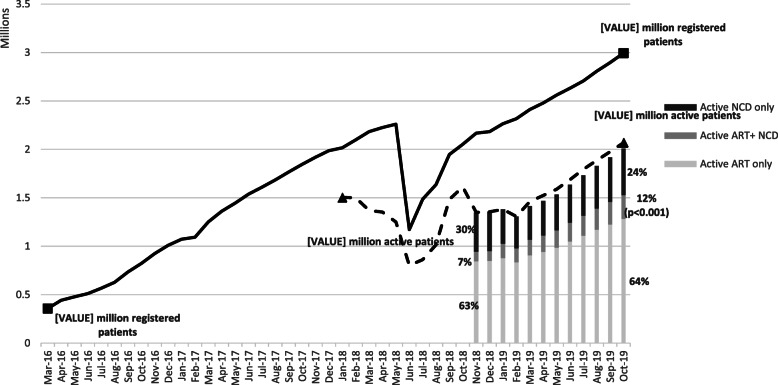
Number of Registered and Active CCMDD Patients from March 2016 through October 2019, and distribution of active patients by medication type; from November 2018 through October 2019*

As the numbers of facilities and external PuPs increased, so did the number of patients served by CCMDD (Fig. 2). The number of registered patients has expanded over 8-fold (Fig. 2), reaching a total of 2,993,044 registered patients and 2,069,039 active patients (69.1 % of registered patients were active) by October 2019.

As more granular data on patients’ medication was recorded by CCMDD, it was found that roughly two-thirds of all active patients were receiving ART only and that, there has been a significant increase from 7 to 12 % in the proportion of patients who are receiving both ART and medications for other chronic conditions through CCMDD (*p* < 0.001). As of October 2019, the majority of patients (76 %) were collecting either ART only or ART in combination with medications for chronic conditions (*n* = 1,529,052), with 24 % or 479,120 patients collecting medications for noncommunicable diseases only. Over time, the number of active patients collecting for each type of medication, i.e., those receiving ART only, ART in combination with medications for chronic conditions (ART NCD), and noncommunicable diseases only (NCD only), increased significantly (*p* < 0.001).

**Fig. 3:**
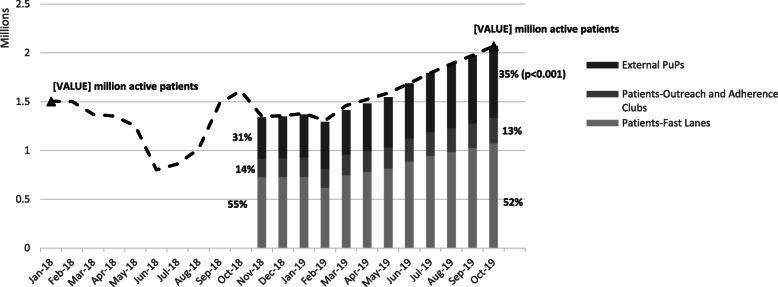
Number and proportion of active CCMDD patients selecting to receive their medication at external PuPs, outreach and adherence clubs, and clinic-based fast lanes; from November 2018 through October 2019*

As shown in Fig. 3, all three types of medication distribution sites (external PuPs, outreach and adherence clubs, and clinic fast lanes) experienced significant increases in patient volume over time (*p* < 0.001). In the year between November 2018 and October 2019, the proportion of active patients relying on clinic-based fast lanes decreased from 55 to 52 % while the proportion using external PuPs increased from 31 to 35 % (*p* < 0.001). By the end of the study period, 734,005 active CCMDD patients were collecting from external pick-up points, a 73 % increase in patient volume to those locations for the year.

## Discussion

We describe growth toward national scale for South Africa’s CCMDD program during Project Last Mile’s support. CCMDD is designed to improve access to medications and retention in care for patients with stable, chronic disease while decongesting public health facilities. From March 2016 through October 2019 (3 years, 7 months), we observed significant increases in the number of districts participating in the program (reaching full saturation in 8 of South Africa’s 9 provinces in January 2018), the number of public health facilities enrolled; the number of external pick-up points registered; and the number of patients engaged. To place this growth in context, by the end of March 2019 [per District Health Barometer 2018/2019] [26], there were 2,850,325 adults on ART from participating districts who were virally suppressed, of which 1,068,938 or ~ 37.5 % were actively enrolled in CCMDD.

As the CCMDD program’s administrative processes and operational systems strengthened, more detailed insights into specific areas of growth were gained (i.e., proportion of active patients electing each type of pickup point, proportion of active patients receiving each medication type). The proportion of active patients who received medication for noncommunicable diseases other than HIV (either alone or in combination with ART) increased during the last year of the reported period, with approximately one out of four patients collecting medications for non-communicable diseases only. This trend highlights the potential of the program in offering integrated NCD and HIV treatment [27–29], a global priority. In particular, these data demonstrate the success of the program in engaging all patients including those who are not living with HIV, which bodes well for the potential of the program to address the rising burden of NCDs in South Africa [30] and serves to help overcome any stigma associated with differentiated service delivery models being labeled as HIV-only programs [31].

Patients’ choice of pick-up points evolved over time, and there were increasing numbers and proportions of patients opting to collect their medications at external PuPs, particularly as more of these external PuPs were contracted. This is encouraging because external PuPs provide the most benefit to the health system, patients and private sector partners, affording patients access to medications in locations convenient to them. This is aligned with Project Last Mile’s strategic and technical inputs (geomapping to inform placement of external PuPs, engagement of private sector partners across the country, and innovation in new external PuP models (e.g. SmartLockers). We also observed growth in the number of patients choosing to collect their medications at outreach and adherence clubs, as well as clinic-based fast lanes, demonstrating the continued value of these options.

At the end of the observation period, nearly 70 % of patients ever registered with CCMDD were considered active. This proportion signals relatively high levels of retention within the program, but also highlights the need for future research to understand the experiences of the remaining 30 % of registered patients who were no longer active in the program.

Our findings should be interpreted in light of their limitations. First, the trends described herein are based on analysis of routine administrative data extracted from government systems and subject to the realities of implementation. The systems for tracking CCMDD expansion became more robust over time (as evidenced by increasingly granular data in later periods). Additionally, in April 2018, new contracts were awarded to two Service Providers and a transition period ensued until September 2018, which included establishing data collection processes at the newly appointed service providers, and integrating and aligning datasets from newer and older service providers. The apparent dip in the number of cumulative and active patients during this time was driven by challenges in transitioning patient records and aligning counts provided by these various service providers. We chose to include the dip, rather then omitting data from the transition months, in order to show the “messiness” of real-world implementation. It is worth noting the tremendous amount of coordination that occurs between the private service providers and the public health system [32], and more robust tools were introduced across service providers after this transition to ensure consistent reporting. Because we report on trends over 43 months of data collection, we remain confident in the overall growth trends observed. Second, this study evaluates expansion of the program from the national level, does not explore district- or provincial-level variation or urban/rural distinctions in uptake of the CCMDD program. Further analysis to understand this variation could be useful to those seeking to strengthen CCMDD implementation and those working toward national scale of DSD models in other settings. Third, although qualitative studies have demonstrated the value of CCMDD to patients [22], our study does not assess whether or how CCMDD expansion contributed to patient-level clinical outcomes, pursuit of the 95-95-95 benchmarks, or more proximal metrics associated with clinic decongestion including staffing, patient volume at clinics, and health care worker satisfaction. However, by the end of March 2019 [per District Health Barometer 2018/2019] [26], there were 2,850,325 adults on ART from participating districts who were virally suppressed, of which 1,068,938 or ~ 37.5 % were actively enrolled in CCMDD, indicating that the CCMDD program had grown to be an important part of the national ART strategy. We hope that the results of our analysis will inspire future studies to evaluate and compare strategies to improve progress toward the UNAIDS 95-95-95 benchmarks. Fourth, data on the demographics of patients participating in the CCMDD program (gender, age, ethnicity) would assist to understand equitable implementation of the program. It should also be noted that the observation period for this data preceeds the Dolutegravir (DTG) rollout (launched December 2019) and changes in CCMDD eligibility criteria to engage patients on ART with viral suppression at six months instead of 12-months (revised March 2020), both of which may have potential impact on CCMDD uptake and retention [33]. Notably, decentralizing routine medication collection, particularly for patients living with chronic disease who may be vulnerable to infectious diseases, has become of increasing importance as health systems adapt to the global threat of the COVID-19 pandemic [32, 34–37].

## Conclusions

In the context of South Africa’s impressive commitment to ensure a healthy life for all which includes “universal test and treat” [6] for people living with HIV and need to address an increasing burden of NCDs [7, 8], and in response to global demand for DSD models that can work at scale [17–19], this longitudinal description of the CCMDD program describes possible path to scale to a national differentiated service delivery model, initially piloted in a small number of districts, that now reaches 88 % of all South African health districts and ~ 95 % of facilities in those districts. This quantification of expantion of CCMDD’s reach, supported by proactive integration of private sector business expertise through the NDoH and PLM partnership [25, 38], will be useful to practitioners, implementers, and policymakers considering mechanisms to expand or establish chronic medication dispensing models, particularly in resource-constrained settings with high patient volume and clinical staff shortages, as well as to researchers evaluating and comparing DSD models within and across country settings. Our results also signal strong potential for integrated DSD models that integrate HIV care with NCD care a key priority for countries that are battling multiple diseases in parallel [25, 38].

## Data Availability

The data that support the findings of this study are available from the authors upon request and with written permission from South Africa’s National Department of Health.

## Abbreviations

ART: Antiretroviral Therapy
CCMDD: Central Chronic Medicines Dispensing and Distribution program
GHLI: Global Health Leadership Initiative
HIV: Human Immunodeficiency Virus
NDoH: National Department of Health
NCD: Non-Communicable Disease
NHI: National Health Insurance
PLM: Project Last Mile
PuP: Pick-up Point
SDG: Sustainable Development Goals
UN: United Nations
USAID: United States Agency for International Development
