# Correction to: Expansion of a national differentiated service delivery model to support people living with HIV and other chronic conditions in South Africa: a descriptive analysis

**DOI:** 10.1186/s12913-021-06561-7

**Published:** 2021-06-04

**Authors:** Lingrui Liu, Sarah Christie, Maggie Munsamy, Phil Roberts, Merlin Pillay, Sheela V. Shenoi, Mayur M. Desai, Erika L. Linnander

**Affiliations:** 1 Global Health Leadership Initiative, Yale School of Public Health, New Haven, USA; 2 Department of Health Policy and Management, Yale School of Ptublic Health, New Haven, USA; 3 National Department of Health, Pretoria, South Africa; 4 Project Last Mile, Pretoria, South Africa; 5 Department of Medicine, Yale School of Medicine, Section of Infectious Diseases, AIDS Program, New Haven, USA; 6 Department of Chronic Disease Epidemiology, Yale School of Public Health, New Haven, USA

**Correction to: BMC Health Serv Res 21, 463 (2021).**

**https://doi.org/10.1186/s12913-021-06450-z**

Following the publication of the original article [1], the authors identified typesetting errors in Fig. 1, Fig. 2 and Fig. 3, the starting and ending values for each of the line charts have been replaced by a [VALUE] placeholder.

**Fig. 1:**
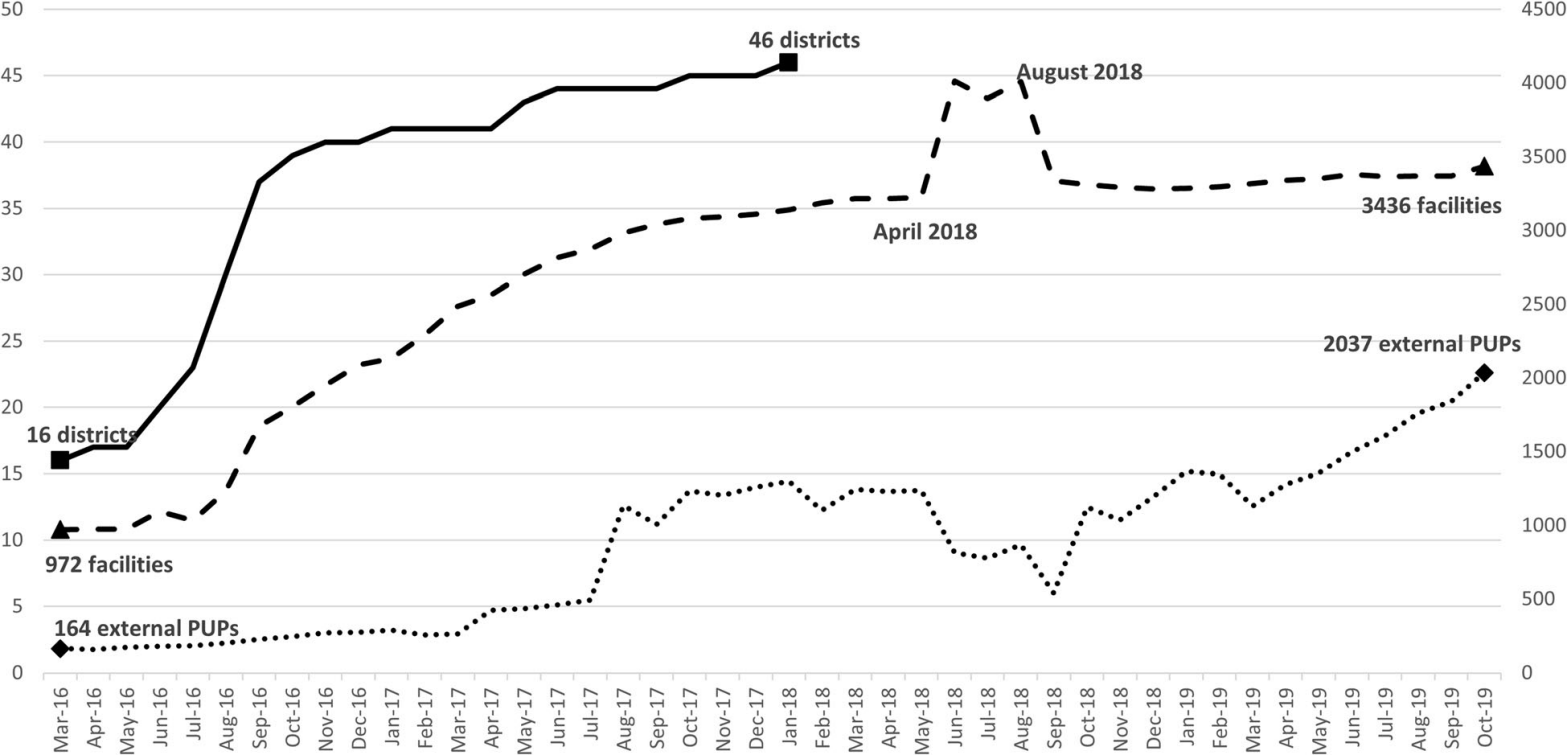
Number of Districts, Facilities, and External Pickup Points (external PuPs) Registered in the Central Chronic Medicines Dispensing and Distribution (CCMDD) Programme; from March 2016 through October 2019*

**Fig. 2:**
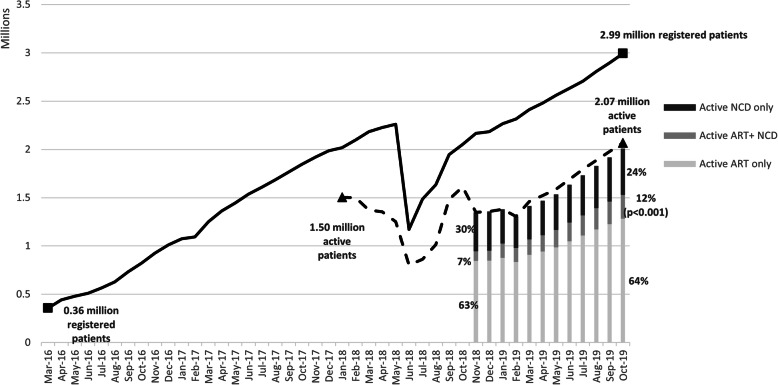
Number of Registered and Active CCMDD Patients from March 2016 through October 2019, and distribution of active patients by medication type; from November 2018 through October 2019*

**Fig. 3:**
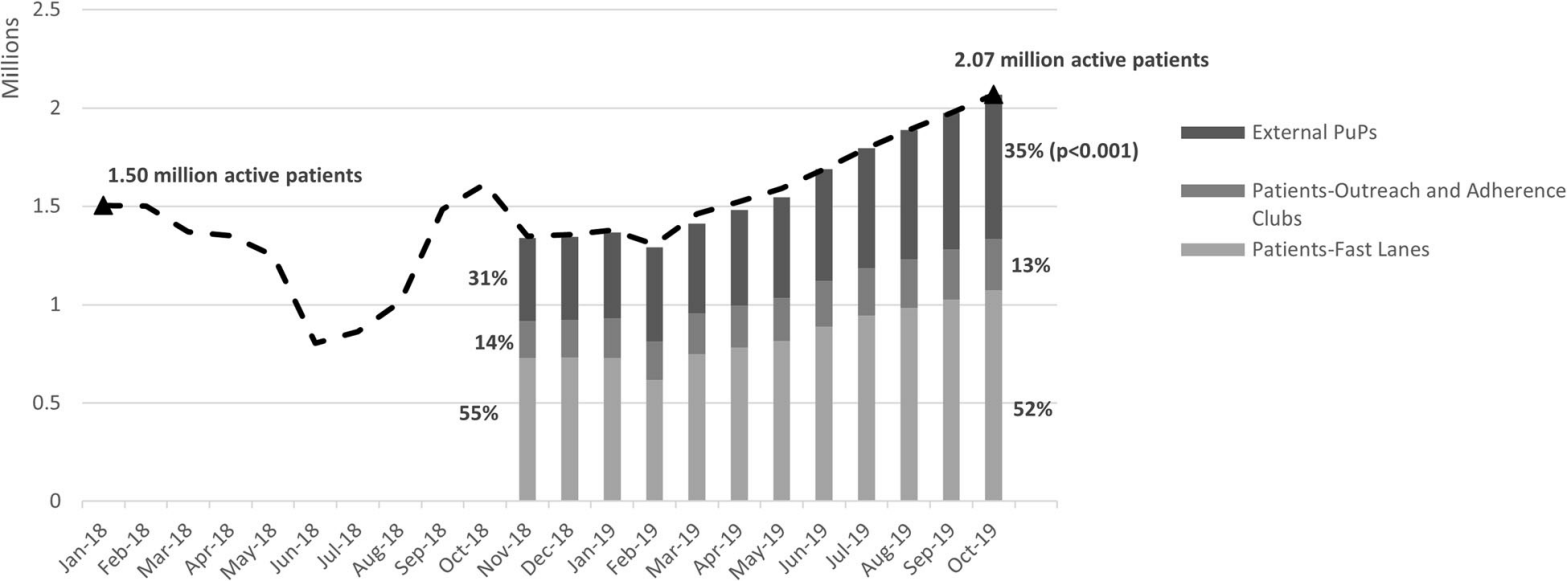
Number and proportion of active CCMDD patients selecting to receive their medication at external PuPs, outreach and adherence clubs, and clinic-based fast lanes; from November 2018 through October 2019*

In addition, the “Title:” should be removed from the article title. The correct article title is: Expansion of a national differentiated service delivery model to support people living with HIV and other chronic conditions in South Africa: a descriptive analysis

The correct figures have been included in this correction, and the original article has been corrected.
